# CD109 is identified as a potential nasopharyngeal carcinoma biomarker using aptamer selected by cell-SELEX

**DOI:** 10.18632/oncotarget.10530

**Published:** 2016-07-11

**Authors:** Wenting Jia, Caiping Ren, Lei Wang, Bin Zhu, Wei Jia, Menghui Gao, Fei Zeng, Liang Zeng, Xiaomeng Xia, Xiaobing Zhang, Ting Fu, Shasha Li, Can Du, Xingjun Jiang, Yuxiang Chen, Weihong Tan, Zilong Zhao, Weidong Liu

**Affiliations:** ^1^ Cancer Research Institute, Collaborative Innovation Center for Cancer Medicine, Key Laboratory for Carcinogenesis of Chinese Ministry of Health, School of Basic Medical Science, Central South University, Changsha, Hunan, P. R. China; ^2^ Department of Gynecology and Obstetrics, The Third Xiangya Hospital, Central South University, Changsha, Hunan, P. R. China; ^3^ Hunan Cancer Hospital and the Affiliated Cancer Hospital of Xiangya School of Medicine, Central South University, Changsha, Hunan, P. R. China; ^4^ Department of Gynaecology and Obstetrics, The Second Xiangya Hospital, Central South University, Changsha, Hunan, P. R. China; ^5^ Molecular Science and Biomedicine Laboratory, State Key Laboratory for Chemo/Bio Sensing and Chemometrics, College of Biology, College of Chemistry and Chemical Engineering, and Collaborative Research Center of Molecular Engineering for Theranostics, Hunan University, Changsha, Hunan, P. R. China; ^6^ Department of Neurosurgery, Xiangya Hospital, Central South University, Changsha, Hunan, P. R. China; ^7^ Hepatobiliary & Enteric Surgery Research Center, Central South University, Changsha, Hunan, P. R. China

**Keywords:** aptamer, cell-SELEX, nasopharyngeal carcinoma, biomarker discovery, CD109

## Abstract

Nasopharyngeal carcinoma (NPC) is one of the most prevailing cancers in southern China and southern Asia. Because of the nonspecific symptoms and lack of effective biomarker, most patients are diagnosed at advanced stages, resulting in poor 5-year survival rate. To identify a novel NPC biomarker facilitating early detection and effective therapy of NPC, a two-step strategy consisting of cancer cell-Systematic Evolution of Ligands by EXponential enrichment (cell-SELEX) procedure and aptamer-based purification approach was developed. Using cell-SELEX procedure, four aptamers (S3, S5, S12 and S27) differentiating the molecular differences between NPC cells and NP cells were successfully screened. Then, using aptamer-based protein purification, membrane protein CD109 was identified as the target of aptamer S3. CD109 protein was further identified to be over-expressed in NPC cell lines and clinic tissues, but not or low in NP cell line and clinic NP tissues, detected by western blot and immunohistochemistry experiments. Our study demonstrated that CD109 identified by cell-SELEX and aptamer-based purification strategy might be used as a potential NPC biomarker for early diagnosis and targeted therapy.

## INTRODUCTION

Nasopharyngeal carcinoma (NPC) is a squamous-cell carcinoma that occurs in the epithelia lining of nasopharynx [1]. NPC is endemic in Southern China and Southeast Asia [2, 3]. The prognosis for patients with NPC depends on the stage of the disease at diagnosis [4]. However, due to minimal or non-specific local symptoms and the relative inaccessibility of the nasopharynx for routine examination, most NPC patients are diagnosed at advanced stage, resulting in poor prognosis and low survival rate [5]. Based on the close relation between NPC and Epstein-Barr Virus (EBV), EBV DNA in plasma or serum has been suggesting as a marker for diagnosis of primary NPC, monitoring of disease relapse and screening of the high-risk population over the past 14 years [6]. However, low reproducibility of quantitative plasma EBV DNA results among different laboratories has limited its clinical applications [5]. Therefore, discovering biomarkers for early diagnosis of NPC becomes critical to improve the overall survival of NPC patients.

Cell membrane proteins play key roles in various physiological functions. Dysregulation and abnormal expression of membrane proteins usually take place in cancer cells. Thus, membrane proteins provide a giant pool of potential disease biomarkers for diagnosis, therapy and prognosis. So far, a panel of differentially expressed proteins has been identified for NPC by mass spectrometry-based proteomic approaches [7]. However, these approaches are time-consuming and usually accompanied with a high ratio of false positives [8]. Moreover, the identification of low-abundance proteins in complex sample with wide dynamic range of abundance of membrane proteins, such as cell lysate, remains a formidable challenge for proteome analysis [9]. To address the challenge, several strategies including affinity-based depletion, ultracentrifugation, and enrichment of target of interest have been developed to reduce the dynamic range of protein concentrations and increase the number of proteins of interest for biomarker discovery [10]. However, these strategies usually suffer from low reproducibility and specificity.

Aptamers, often termed chemical antibodies, have attracted extensive attention as novel molecular probes in bioanalysis and biomedicine. Aptamers are single-stranded oligonucleotides screened from RNA/DNA library through an *in vitro* iterative selection process known as Systematic Evolution of Ligands by EXponential enrichment (SELEX) [11, 12]. They can bind to their targets with high affinity and high specificity by folding into distinct tertiary structures. Aptamers can be selected to recognize purified cancer-related proteins such as VEGF [13], PDGF [14], even mutant EGFRvIII [15]. In addition, by applying cell-SELEX, aptamers can be generated to recognize the molecular signatures of a given cell phenotype, even to differentiate the molecular difference between cancer cells and normal cells without prior knowledge of molecular signatures [16–19]. Aptamers are also easy to be chemically modified and conjugated with various matrices to separate and enrich target proteins from complex samples with a wide dynamic range of protein abundance for proteome analysis. Thus, cell-SELEX provides powerful and effective molecular tools for new biomarker identification. Up to now, by using a two-step consisting of cell-SELEX and aptamer-based affinity purification, combined with mass spectrometry, many membrane protein biomarkers such as tenascin C [20], protein tyrosine kinase 7 [21], stress-induced phosphoprotein 1 [22], alkaline phosphatase placental-like 2 [23], selectin L and integrin α4 [24] have been identified for diagnosis and therapeutics.

In this study, by using cell-SELEX approach with NPC 5-8F as target cells and nonmalignant human nasopharyngeal (NP) epithelial NP69 cells as negative control cells, four aptamers (S3, S5, S12 and S27) that can differentiate the molecular differences between NPC cells and NP cells were identified. By aptamer-based affinity purification, combined with mass spectrometry, CD109 was identified as the target of aptamer S3. The fact that CD109 is expressed on the cell surface of NPC cell lines and clinical NPC tissue specimens, but no or lowly expressed in NP cell line and clinic NP tissues, makes it an attractive target for early diagnosis and therapy of NPC.

## RESULTS

### Selection of aptamers against NPC cells

**Figure 1:**
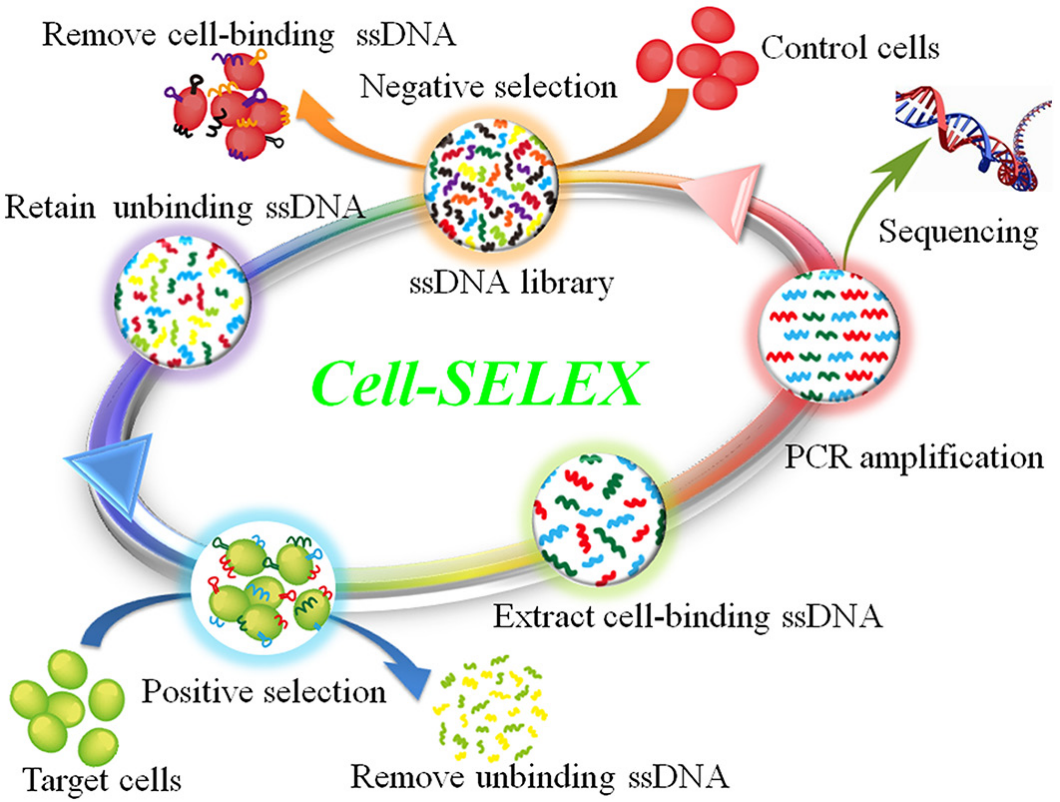
Scheme of cell-SELEX against NPC 5-8F cell line The ssDNA library was incubated with nonmalignant NP69 cells to remove nonmalignant NP cells-binding ssDNAs. The unbound ssDNAs were then incubated with NPC 5-8F cells for positive selection. After washing, the bound ssDNAs were eluted and amplified by PCR for next-round selection. The evolved ssDNA pool was sequenced to identify individual aptamer sequences after 25 rounds of enrichment.

To generate NPC cells-recognizing aptamers, a cell-SELEX process was directed against NPC 5-8F cell line, with nonmalignant NP69 cell line as negative control. The scheme of cell-SELEX process was illustrated in Figure 1. In first-round selection, the initial single-stranded DNA (ssDNA) library was only applied on NPC 5-8F cell monolayer for positive selection. From the second round of selection, the evolved ssDNA library was first incubated with NP69 cells to remove nonmalignant NP cell-binding ssDNA, and then the unbound ssDNA was collected and incubated with target 5-8F cells for positive selection.

**Figure 2:**
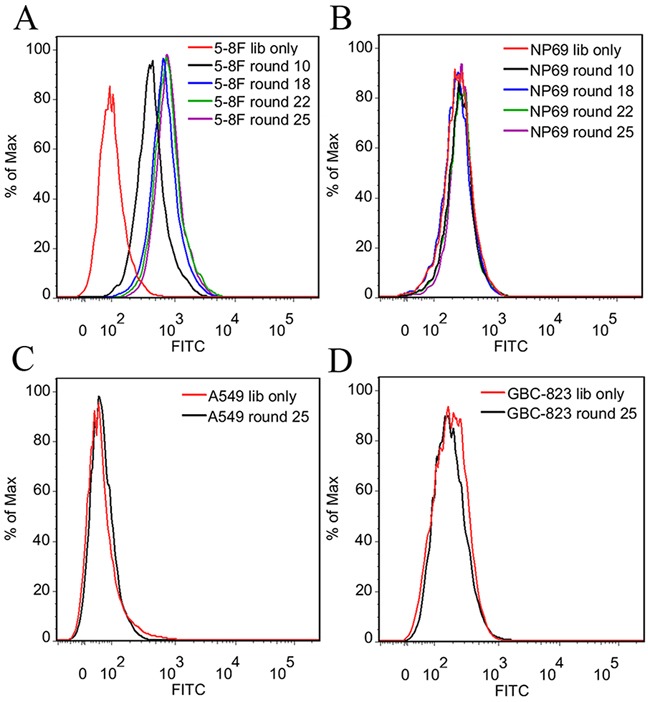
Flow cytometry analysis of the binding ability of the evolved ssDNA pools **A.** The fluorescence intensity of 5-8F cells was gradually increased after incubation with increasing rounds of ssDNA pools. **B.** No increase in fluorescence signal was observed for negative control NP69 cells after incubation with ssDNA pools. No obvious binding on lung adenocarcinoma A549 cells **C.** and gastric carcinoma GBC823 cells **D.** were observed after incubation with the 25^th^ round ssDNA pool. It implied that the selected DNA pool might specifically bind to NPC cells. The unselected initial ssDNA library was used as a control.

During selection, the enrichment process of target cell-binding ssDNA was monitored by flow cytometry. As shown in Figure 2, target 5-8F cells presented sharp increases in fluorescence intensity after incubation with FITC-labeled ssDNA pools from the first 18 rounds of selection. However, there were very slight changes in fluorescence intensity of 5-8F cells after incubation with ssDNA pools from the next 7 rounds of selection. The results indicate that the target cell-binding DNA sequences have been gradually enriched during selection, and the enrichment process finished at about the 22^nd^ rounds of selection. In addition, almost no increase in fluorescence signal was observed for negative control NP69 cells after incubation with FITC-labeled evolved ssDNA pools (Figure 2B). The results demonstrated that the negative selection was very effective to remove nonmalignant NP cell-binding ssDNA in this study. Moreover, the evolved ssDNA pool from the 25^th^ round of selection had little binding on lung adenocarcinoma A549 cells and gastric carcinoma GBC823 cells (Figure 2C and 2D), implying that the selected DNA pool might specifically bind to NPC cells.

### Binding specificity analysis of selected aptamers

**Figure 3:**
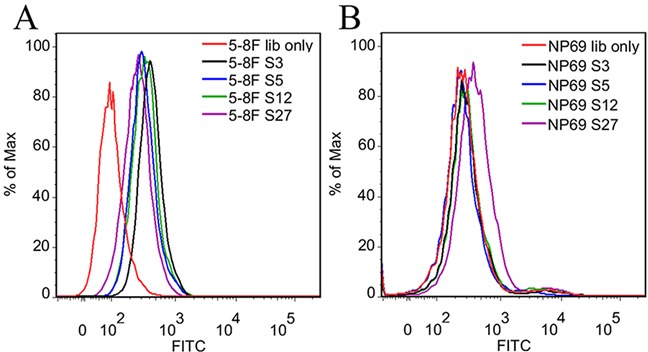
Flow cytometry analysis of the binding ability of the selected four DNA aptamers The unselected initial ssDNA library was used as control. **A.** The fluorescence intensity of selected aptamers bound to target 5-8F cells indicated all of them could bind to 5-8F cells. **B.** The fluorescence intensity of selected aptamers bound to NP69 cells indicated they did not bind to NP69 cells.

The highly enriched aptamer pool from the 25^th^ round of selection was cloned and sequenced (Supplementary Figure S1). The sequences from 100 clones were grouped based on the homology of random sequences. Seven representative aptamer candidates from the most abundant 7 groups were chosen and their binding ability on target cells was examined by flow cytometry. As shown in Figure 3, four aptamers (S3, S5, S12 and S27, Table 1) could bind to target NPC 5-8F cells. Among the four aptamers, S3, S5 and S12 had little binding on negative control NP69 cells, only S27 had weak binding on NP69 cells.

**Table 1:** Aptamer sequences recognizing NPC 5-8F cells

Name	Sequence
S3	atccagagtgacgcagcaTCTGAGAATAGTGGTTTGCTGTATGGTGGGCGTTGAAAGAGGGGtggacacggtggcttagt
S5	atccagagtgacgcagcaCTGTGGCGGGATTCTGGCAAAGTTTCGAGCCCTGGTAAGAGTGtggacacggtggcttagt
S12	atccagagtgacgcagcaCGCCGTAGTATGGTGCAGATGGTTTGCTGTATGGTGGGCGCACCtggacacggtggcttagt
S27	atccagagtgacgcagcaGAAAGTAAGGGTAGTTTGGGGGCTCGTATGGGGGGAGGTTCTGAtggacacggtggcttagt

The binding ability of the four aptamers on other cell lines including 10 NPC cell lines, 7 other cancer cell lines and one immortalized cell line was further assayed by flow cytometry. As shown in Table 2 and Supplementary Figure S2, aptamer S3 could recognize all the tested NPC cell lines, and aptamer S5, S12 and S27 could recognize 7, 6 and 9 cell lines out of the tested 10 NPC cell lines, respectively. In addition, aptamer S3, S5 and S12 could not recognize the immortalized normal human embryonic kidney HEK293T cell line. Among the four aptamers, S3 showed the best specificity for NPC cell lines, and could not recognize 5 cell lines out of the tested 7 other cancer cell lines. These results indicate that the aptamers selected in this cell-SELEX could be used as molecular probes to profile the differences between NPC cell lines and normal NP cell line at molecular level.

**Table 2:** Recognition of aptamers on different cell lines

NPC cell line	S3	S5	S12	S27
5-8F	++++	++++	++++	+++
6-10B	+++	+++	++++	++
CNE1	+	0	0	ND
CNE2	+	0	0	+
HNE1	+++	+++	+++	++++
HNE2	++++	++++	++++	++++
HNE3	+	+	0	++
HONE1	+	++	++	++++
SUNE1	++	++++	++++	++++
C666-1	+	0	0	++
HK-1	+	+	+	++++
Other cell lines				
A549	0	0	+	0
GBC-823	0	0	+	++
MG63	0	ND	ND	0
HRT-18	0	0	0	ND
U87	0	ND	ND	ND
Caski	++	ND	ND	ND
Hela	++	ND	ND	+
HEK-293T	0	0	0	++
NP69	0	0	0	++
WBC	0	ND	ND	ND

A threshold based on fluorescence intensity of FITC in the flow cytomery analysis was chosen so that 99% of cells incubated with the FITC-labeled unselected DNA library would have fluorescence intensity below it. When the FITC-labeled aptamer was allowed to interact with the cells, the percentage of the cells with fluorescence above the set threshold was used to evaluate the binding capacity of the aptamer to the cells. 0, <10%; +, 10–35%; ++, 35–60%; +++, 60–85%; ++++, >85%; ND: not detected.

### Imaging tumor cell sphere with aptamer S3

**Figure 4:**
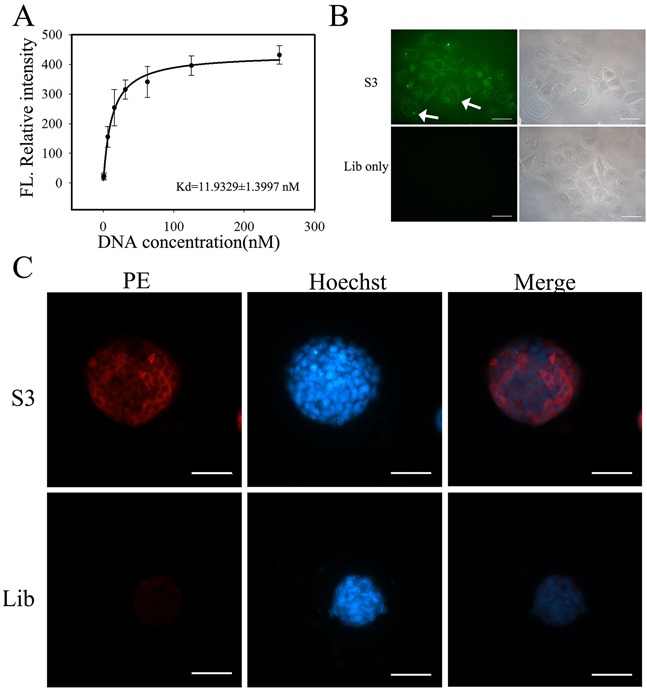
Binding ability of aptamer S3 to NPC 5-8F cells **A.** Dissociation constant of aptamer S3 on target NPC 5-8F cells. **B.** Fluorescence imaging of NPC 5-8F cells stained by FITC-labeled S3 or library. (Scale bar, 50 μm) **C.** Fluorescence imaging of 5-8F cells formed-NPC tumor spheres by incubation with biotin-labeled S3 or library followed by the incubation of streptavidin-PE. Biotin-labeled unselected DNA library was used as negative control. (Scale bar, 100 μm)

Since aptamer S3 presented best NPC cells-specific binding ability than the other three, it was chosen for further investigation. As shown in Figure 4A and 4B, aptamer S3 could specifically recognize 5-8F cells with an equilibrium dissociation constant (*K_d_*) of 11.93 ± 1.40 nM. The recognition ability of aptamer S3 on 5-8F tumor sphere was also tested by fluorescence microscopy. After incubation with biotin-labeled aptamer S3 followed by incubation of streptavidin-PE, NPC 5-8F tumor spheres presented bright red fluorescence. However, NPC 5-8F tumor spheres only presented very weak red fluorescence after incubation with biotin-labeled initial library and strepavidin-PE (Figure 4C).

### Effect of incubation temperature on binding ability of S3

**Figure 5:**
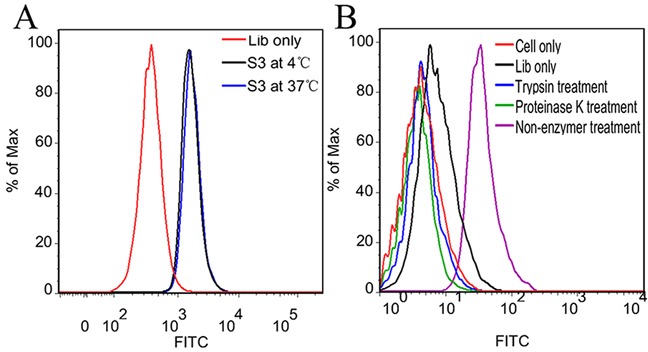
Effect of incubation temperature and proteinase treatment on binding ability of S3 **A.** Flow cytometric analysis of the FITC-labeled aptamer S3 on 5-8F cells at 4°C or 37°C. **B.** Flow cytometric analysis of the FITC-labeled aptamer S3 on 5-8F cells treated with trypsin or proteinase K.

The aptamer selection procedure was performed at 4°C to avoid enriching nonspecific DNA sequences caused by endocytosis at 37°C. However, selection at 4°C might generate aptamers with poor binding ability at physiological temperature [20, 25]. To test whether the change in incubation temperature would damage the binding ability of aptamer S3, its binding ability on NPC 5-8F cells at different incubation temperature was analyzed by flow cytometry. As shown in Figure 5A, NPC 5-8F cells presented almost the same fluorescence signals after incubation with aptamer S3 at 4°C or 37°C, suggesting that the incubation temperature has little effect on the binding ability of S3.

### Target type analysis of aptamer S3

To determine whether the binding target of S3 is an extracellular membrane protein, the binding affinity of S3 on NPC 5-8F cells treated with trypsin or proteinase K for 5 min was investigated. As shown in Figure 5B, aptamer S3 lost its binding ability to these cells, whose membrane proteins were destroyed by trypsin or proteinase K. The results suggest that the binding targets of S3 are most likely to be extracellular proteins.

### Recognition of NPC cells in peripheral blood using FITC-labeled S3

**Figure 6:**
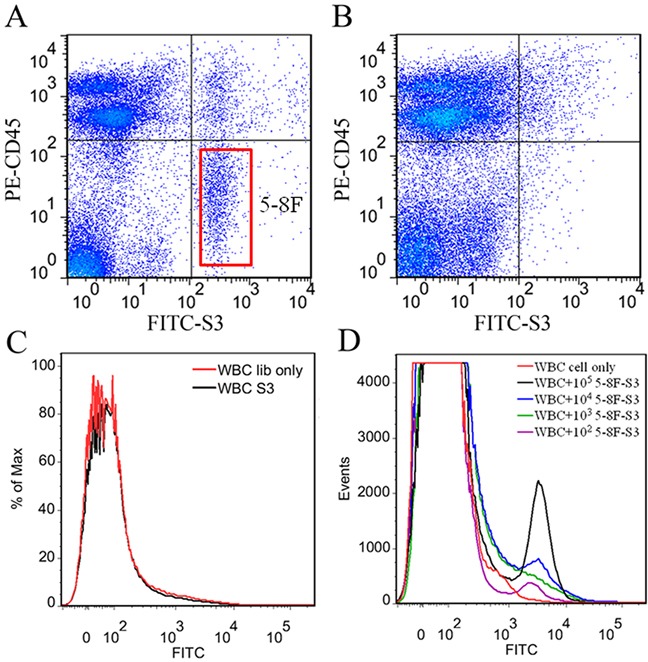
Recognition of the target cells in real peripheral blood samples by S3 **A.** 10^5^ NPC 5-8F cells dispersed in 1mL of peripheral blood from healthy volunteer. **B.** 10^5^ A549 cells dispersed in 1mL of peripheral blood from healthy volunteer. PE-labeled anti-CD45 monoclonal antibody was used to recognize the white blood cells (WBCs). **C.** Flow cytometric analysis of aptamer S3 with WBCs. **D.** Flow cytometric analysis for the binding of the FITC-labeled aptamer S3 with 10^5^, 10^4^, 10^3^ and 10^2^ 5-8F cells dispersed in 1 mL of peripheral blood.

It is very important for aptamers to retain their recognition ability in complex physiological environment. To test the feasibility of using aptamer S3 as a specific probe to recognize NPC cells in blood, 10^5^, 10^4^, 10^3^ and 10^2^ 5-8F cells or 10^5^ A549 cells were dispersed in 1 mL of peripheral blood from a healthy volunteer, respectively. After lysis of erythrocytes, the suspension containing cancer cells was incubated with FITC-labeled S3 on ice. Flow cytometric analysis demonstrated that aptamer S3 could recognize NPC 5-8F cells (Figure 6A), but not lung adenocarcinoma A549 cells dispersed in 1 mL of peripheral blood (Figure 6B) or white blood cells (WBCs) (Figure 6C). Moreover, it could also be seen that aptamer S3 could detect as few as 10^2^ NPC 5-8F cells in 1 mL of peripheral blood (Figure 6D). These results indicated that aptamer S3 might be used as an effective molecular probe for NPC research.

### Staining clinical NPC tissue section with S3

**Figure 7:**
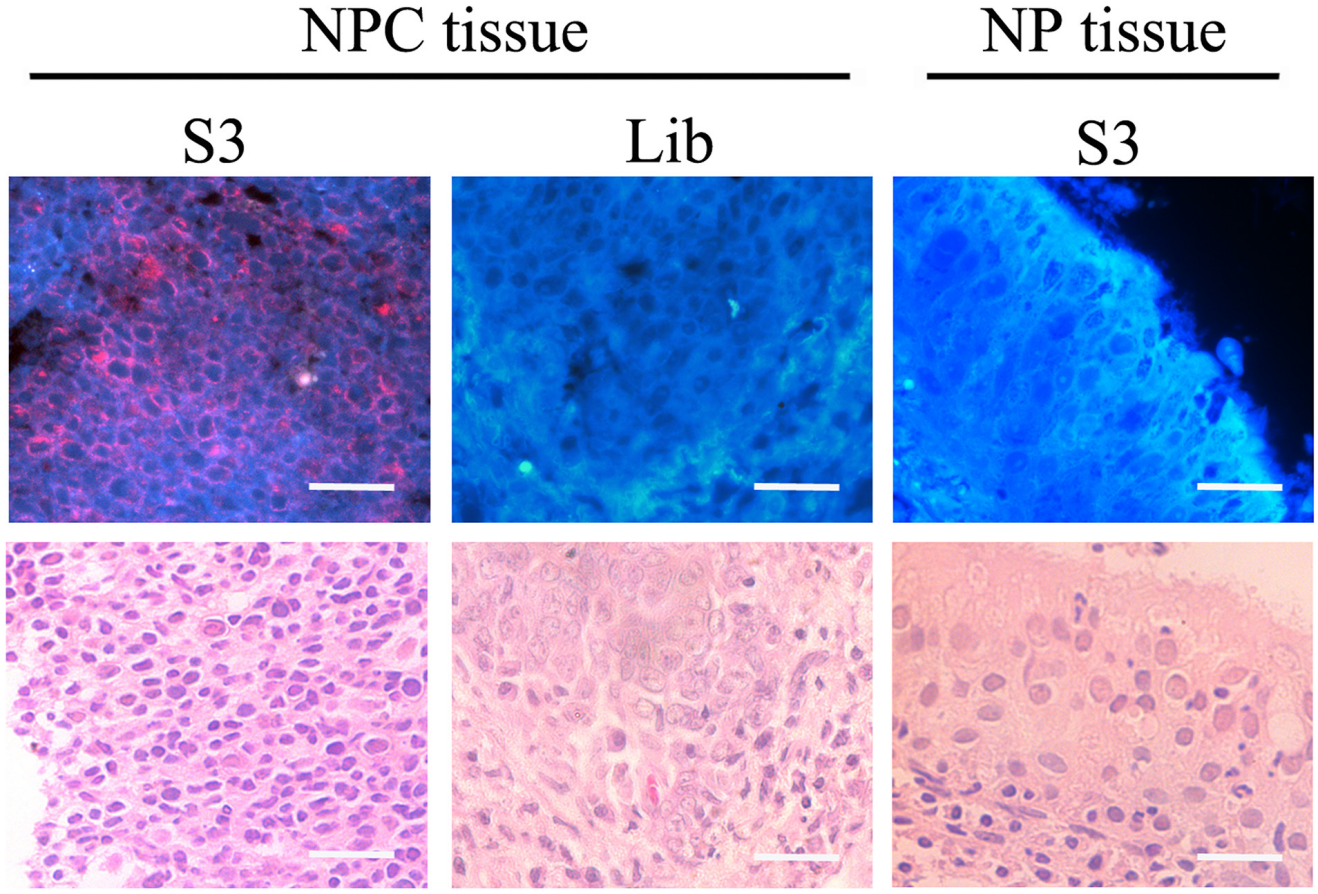
Fluorescence imaging of cancer cells in NPC tissues stained with QD635-labeled S3 aptamer or library Normal NP tissues were used as controls. There were red fluorescence signals in NPC tissues, but no fluorescence in normal NP tissues. NPC tissues presented no fluorescence signal when treated with biotin-labeled ssDNA library and streptavidin-QD625. (Scale bar, 20 μm)

To test whether aptamer S3 could recognize the NPC cells in clinical specimens, formalin-fixed paraffin-embedded (FFPE) NPC tissues and normal NP tissues were incubated with biotin-labeled aptamer S3 followed by incubation with streptavidin-QD625. It could be seen from fluorescence microscopy analysis that there was red fluorescence signal on the cell membrane in NPC tissues, but no fluorescence in normal NP tissues. In addition, NPC tissues presented no fluorescence signal when treated with biotin-labeled ssDNA library and streptavidin-QD625 (Figure 7).

### Identification of the S3 target protein

**Figure 8:**
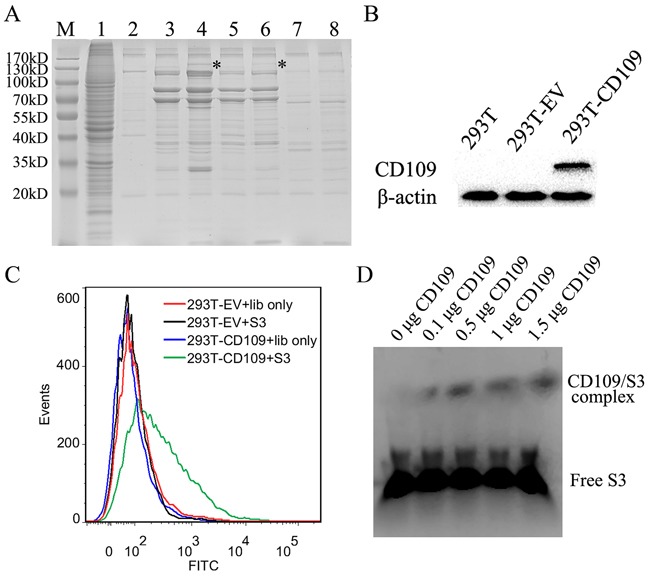
Analysis and verification of the target protein recognized by aptamer S3 **A.** Coomassie brilliant Blue stained SDS-PAGE (12%) was used to analyze the S3-assisted target purification. M, molecular marker; lane 1, 5-8F cell lysate; lane 2, beads only; lane 3, proteins captured with unselected ssDNA library from 5-8F cell lysate; lane 4, proteins captured by aptamer S3 from 5-8F cell lysate; lane 5, proteins captured with unselected DNA library from HNE2 cell lysate; lane 6, proteins captured by aptamer S3 from HNE2 cell lysate; lane 7, proteins captured with unselected DNA library from A549 cell lysate; lane 8, proteins captured with aptamer S3 from A549 cell lysate. **B.** Western blot analysis of the expression of CD109 in native 293T cells and 293T cells transfected with CD109 vector (designated as 293T-CD109 cells) or CD109 gene-free empty vector (EV) (designated as 293T-EV cells). **C.** Flow cytometry analysis of the binding of aptamer S3 to 293T-CD109 cells or 293T-EV cells. Unselected initial ssDNA library was used as a control. **D.** EMSA assay was performed to confirm the binding of S3 to CD109 in vitro.

To verify the target protein of aptamer S3, the lysate of NPC 5-8F cells was incubated with 125 pmol of biotin-labeled aptamer S3. The target protein was then enriched by using strepavidin-coated beads and eluted by boiling target protein-aptamer-bead complex for 5 min in sample buffer at 95°C. The eluted protein was further resolved on a 4-12% gradient gel. Comparing with negative controls, S3-specific protein bands at 170-130 kDa were shown in lane 4 and 6 (Figure 8A). The band was treated with trypsin digestion and analyzed by mass spectrometry (MS). The MS results revealed that membrane protein CD109 might be the target candidate of aptamer S3. To validate CD109 was indeed the target protein of S3, aptamer S3 was incubated with 293T cells and CD109-transfected 293T cells (Figure 8B). It could be seen that aptamer S3 could recognize CD109-transfected 293T cells, but not native 293T cells and 293T cells transfected with CD109 gene-free vector compared with unselected initial ssDNA library (Figure 8C). EMSA experiment was also used to validate that the target protein of S3 was CD109 (Figure 8D). The results illustrated that S3 could bind to CD109 *in vitro*. Thus, our results both *in vivo* and *in vitro* showed that CD109 was the target protein of aptamer S3.

### Expression of CD109 in NPC cell lines, tissues and tumor sphere

**Figure 9:**
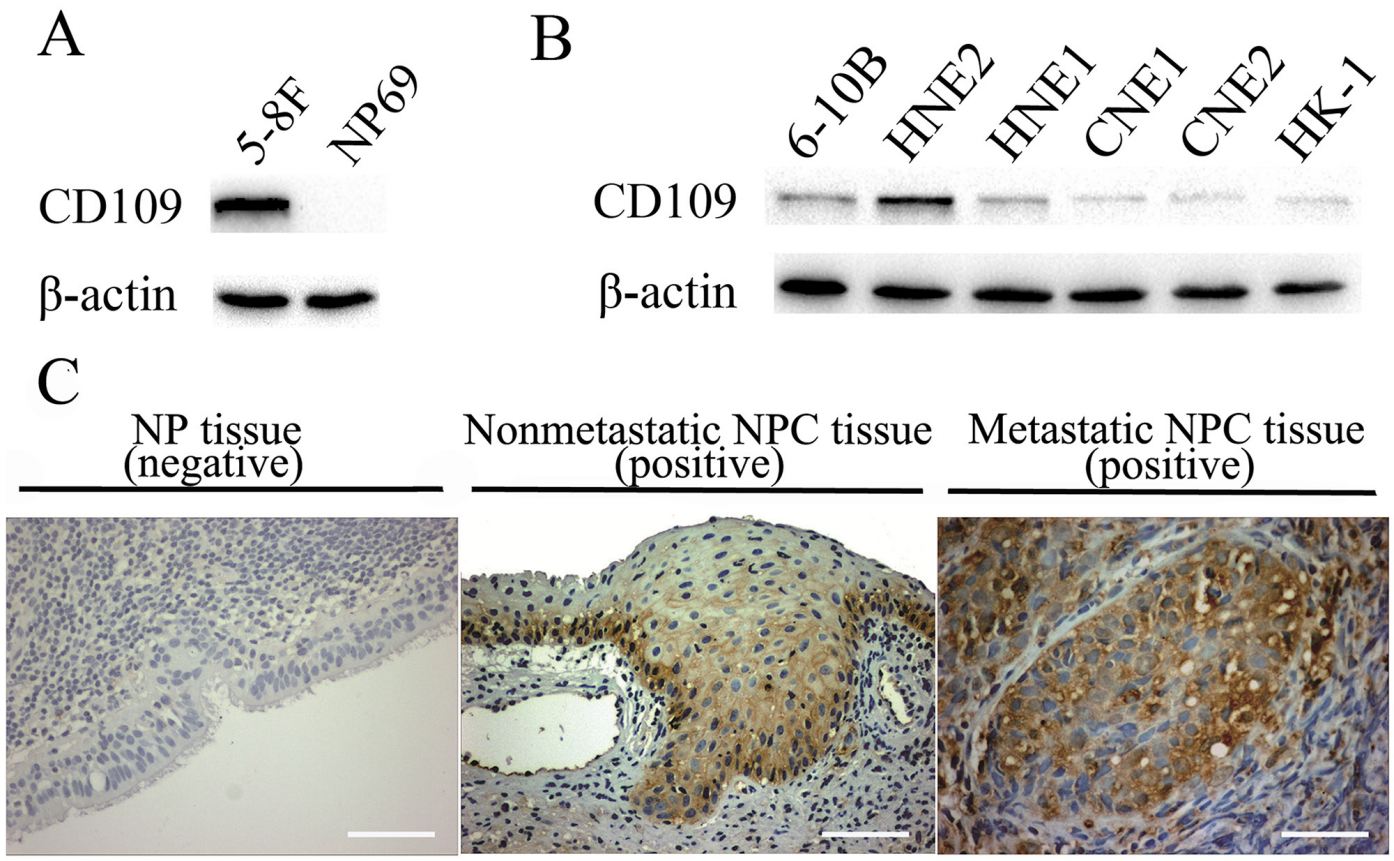
Expression of CD109 in different cell lines and immunostaining of CD109 in clinical NPC and NP tissues **A.** The expression of CD109 in 5-8F and NP69 cells. **B.** The expression of CD109 in 6 other NPC cell lines. CD109 was expressed in all the tested NPC cell lines, but not detectable normal NP69 cells.**C.** Immunostaining of CD109 in clinical NPC and NP tissues. CD109 was positively expressed in both nonmetastatic and metastatic NPC tissues, but it was not detectable or fairly low in NP control tissues. (Scale bar, 100 μm)

CD109 protein expression in 8 NPC cell lines and a normal NP cell line was investigated by western blot experiment. It could be seen that CD109 was expressed in all the tested NPC cell lines, but not detectable in normal NP69 cells (Figure 9A and 9B). The results were well consistent with the results of binding analysis of aptamer S3 (Table 2). For example, 5-8F cells and HNE2 cells, which presented the best fluorescence signal after incubation with FITC-labeled aptamer S3 in flow cytometric analysis, also presented high expression of CD109.

CD109 protein expression in 30 clinical NPC specimens and 12 NP tissues was also analyzed by immunohistochemistry (IHC). As shown in Figure 9C and Table 3, CD109 was positively expressed in all NPC tissues (30 cases), however, it was not detectable (11 cases) or fairly low (1 case) in NP control tissues. Furthermore, we compared the positive expression rate and intensity between NP and NPC tissues, nonmetastatic and metastatic NPC tissues respectively. There existed a statistical difference between NP and NPC tissues, but did not between nonmetastatic and metastatic NPC tissues (Table 3). Thus, CD109 might have close correlation with the early occurrence and development of NPC.

**Table 3:** CD109 protein expression in NPC or NP clinical tissues

Characteristics	CD109 expression				Total	*P* value
	Negative (0)	Weak (1-4)	Moderate (5-9)	Strong (10-15)		
NP (a)	11	1	0	0	12	*P*_a-b_<0.01
Non-metastatic NPC (b)	0	0	5	7	12	*P*_b-c_>0.05
Metastatic NPC (c)	0	0	10	8	18	*P*_a-c_<0.01

**Figure 10:**
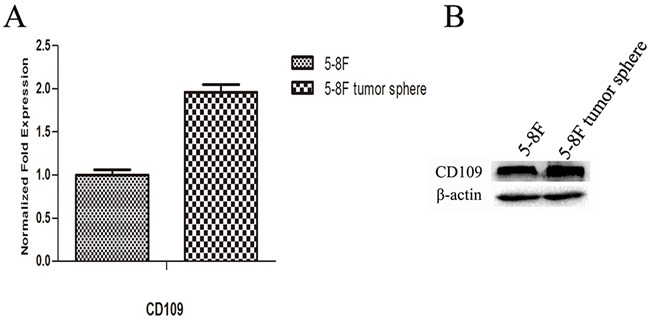
Expression level of CD109 in 5-8F cells and 5-8F tumor spheres mRNA level **A.** and protein level **B.** of CD109 in 5-8F cells and tumor spheres. The results indicated that the expression levels of CD109 were higher in tumor spheres than that in 2D cultured NPC cells, both at mRNA and protein levels.

QPCR and western blot were used to detect the expression levels of CD109 in 5-8F cells and 5-8F tumor spheres. The results indicated that the expression level of CD109 was higher in tumor spheres than that in 2D cultured NPC cells (Figure 10). From the results, we noticed that both NPC CSCs and non-CSCs could express CD109, though NPC CSCs expressed CD109 more strongly.

## DISCUSSION

Carcinogenesis is a multistage process, in which a series of changes in genes, proteins and other molecules are accumulated. These molecules can serve as useful tumor molecule markers. Cancer cell-specific membrane proteins are considered as the most appropriate biomarkers due to the fact the identification of proteins can facilitate understanding tumor progression, developing diagnostic tools, and identifying new therapeutic targets. CD109, a GPI-linked cell membrane protein [26], has been reported to be highly expressed in many types of tumors, such as lung, oral and cutaneous squamous cell carcinomas (SCC), urothelial carcinomas, malignant melanomas, triple-negative breast cancer (TNBC), esophageal squamous cell carcinoma and gallbladder cancer [27–34], but not in most normal human tissues, except for myoepithelial cells of mammary, lacrimal and salivary glands and basal cells of prostate and bronchial epithelia [35]. It was reported that oral SCC with the over-expression of CD109 exhibited accelerated cell growth, and CD109-positive oral dysplastic lesions showed a predisposition to progress to SCC in three years [36]. In addition, a strong correlation was detected between CD109 expression and prognosis in soft tissue sarcomas and TNBC [37]. These data suggest that CD109 expression may be associated with the pathogenesis and prognosis of several human tumors. In this study, CD109 was identified as a target protein of aptamer S3, which can differentiate the difference between NPC 5-8F cells and nonmalignant NP69 cells. CD109 was also identified by Western blot and immunohistochemistry to highly express in NPC cell lines/tissues, but undetectable or fairly low in control NP cell line/tissues. Thus, CD109 may play an important role in early development of NPC and may act as a potential NPC protein biomarker.

Cancer stem cells (CSCs) are a small group of tumor cells with the capacity of self-renewal and differentiation, and the ability to initiate tumorigenesis and maintain ongoing tumor growth. Recently, some researchers suggested that high expression of CD109 regulates the phenotype of cancer stem-like cells/cancer-initiating cells (CSCs/CICs) in the novel epithelioid sarcoma cell line ESX and may be a CSCs/CICs biomarker in epithelioid sarcoma [38]. In another study, Tao et al. also reported that CD109 was over-expressed in breast CSCs [37]. Tumor sphere is one of the methods to enrich CSCs. In the study, we found that CD109-specific aptamer S3, but not unselected ssDNA library, could recognize the tumor spheres. This result indicates that CD109 is not only expressed in NPC cells, but also in NPC CSCs, which was further confirmed by qPCR and western blot. CSCs are the vital factor of malignant tumor recurrence and metastasis. When the drug targets to CD109, it may kill both NPC cells and NPC CSCs, and may have better clinical treatment effects.

NPC has highly invasive and distant metastasis features, and about 90% of patients show cervical lymph node metastasis at the first time of diagnosis [39]. It was found that CD109-specific aptamer S3 could recognize not only metastatic NPC 5-8F cell line, but also non-metastatic NPC 6-10B cell line. Furthermore, CD109 is expressed in both nonmetastatic and metastatic NPC tissues. In simulated peripheral blood environment of NPC patients, aptamer S3 could specifically recognize NPC cells but didn't bind to lung cancer A549 cells, CD45-positive white blood cells and other blood cells. We also found that NPC cells from 1 mL peripheral blood containing 10^2^ 5-8F cells could be identified by the FITC-labeled aptamer S3. These results indicate that aptamer S3 has the capability to capture NPC cells in blood and provides molecular tools for detection of metastatic NPC cells and prognosis analysis of NPC.

In conclusion, compared with traditional proteomic approaches, the two-step strategy consisting of cell-SELEX and aptamer-based affinity purification for biomarker discovery strategy possesses two appealing characterizations. One is that the specific molecular recognition between aptamers and their targets make it possible to achieve the identification of both quantitative and qualitative differences between normal and diseased proteomes. Another is that negative selection with the normal proteome in cell-SELEX and aptamer-based affinity purification facilitates the identification of less abundant biomarkers that are usually missed by most mass-spectrometric techniques due to the presence of more abundant proteins. The aptamer S3, identified by cell-SELEX approach, has good affinity for CD109, and can recognize its target in peripheral blood, tumor spheres, and clinical tissue specimens. Therefore, besides having potential application as a diagnostic tool, aptamer S3 also has straightforward applications in molecular imaging and targeted therapeutics. Moreover, the use of aptamer S3 is not limited to NPC alone as it can be used for all other cancers that have aberrant expression of CD109.

## MATERIALS AND METHODS

### Cell lines and cell culture

All the cell lines used in this study were maintained in our laboratory. NPC cell lines included CNE1, CNE2, HNE1, HNE2, HNE3, SUNE1, HONE1, C666-1 and HK-1, and other tumor cell lines included A549, GBC-823, MG63, HRT-18, U87, Caski and Hela. All of the cell lines were cultured in RPMI1640 containing 10% FBS. NPC 5-8F cell line, which has high tumor formation ability and metastatic potential, was used as target cells. Nonmalignant human NP epithelial NP69, which was cultured in KSFM medium, was used as control cells. Other cell line including human embryonic kidney cell line HEK293T was cultured in DMEM medium containing 10% FBS.

### SELEX library, primers and buffer

The ssDNA library used in the study contained a random sequence of 44 nucleotides flanked by two 18-nucleotide primer-hybridizing sequences for PCR (5′-ATC CAG AGT GAC GCA GCA (44N) TGG ACA CGG TGG CTT AGT-3′). A FITC-labeled sequence (5′-FITC-ATC CAG AGT GAC GCA GCA-3′) and a biotin-labeled sequence (5′-biotin-TGGACACGGTGGCTTAGT-3′) were used as forward primer and reverse primer to amplify the evolved ssDNA pool after each round of selection, respectively. All DNA sequences were synthesized and HPLC-purified by TaKaRa Biotechnology Co. Ltd (Dalian, China).

Washing buffer was prepared with Dulbecco's phosphate buffered saline (D-PBS, Ca^2+^/Mg^2+^-free) supplemented with 4.5 g/L of glucose and 5 mM of MgCl_2_. Binding buffer was prepared with washing buffer added with 0.1 mg/mL of yeast tRNA, and 1 mg/mL of BSA.

### Procedures of cell-SELEX

Cell-SELEX was performed according to previous protocol with minimal modification [40, 41]. The initial ssDNA library (10 OD) dissolved in 1 mL of binding buffer was denatured at 95°C for 5 min, and then cooled on ice. 5-8F cells were washed three times with washing buffer and then incubated with the denatured ssDNA pool on ice for 1 h on a rotary shaker for positive selection. After incubation, the supernatant was discarded and cells were washed with washing buffer. Then 5-8F cells were scraped off and the cell-bound ssDNA sequences were eluted by heating at 95°C for 10 min. After centrifugation, the supernatant was kept and used as a template to prepare evolved ssDNA pool with forward primer and reverse primer by PCR.

The amplified conditions of PCR were as follow: 95°C for 2.5 min, 95°C for 30 s, 56.3°C for 30 s, and 72°C for 30 s, followed by 3 min at 72°C. 10-20 cycles were chosen to minimize the nonspecific amplicons. The FITC-labeled ssDNA were separated from the biotin-labeled complementary sequences by streptavidin-coated sepharose beads (GE Healthcare) after alkaline denaturation (0.2 M NaOH). After being de-salted with NAP-5 column (GE Healthcare), the FITC-labeled ssDNA pool was concentrated and used for next round selection or cytometric analysis.

From the second round of selection, the evolved ssDNA pool was first incubated with NP69 cells in 100-mm culture dish with 90% confluence at 4°C for subtractive selection. After incubation, the supernatant was collected and then applied on 5-8F cells in 100-mm culture dish for positive selection. To acquire aptamers with high affinity and specificity, 10% FBS was added into the binding buffer from the fourth round and gradually increased to 20% in the following rounds. From the fifth round of selection, 5-8F cells were cultured in 60-mm culture dish, and the wash strength was enhanced gradually. At the same time, the positive incubation time was decreased from 1 h to 0.5 h.

After 25 rounds of selection, the selected ssDNA pool was amplified by PCR using unmodified primers, cloned into pMD18-T vector (TaKaRa) and transformed into E. Coli DH-5α. The positive clones were sequenced by BGI Co. Ltd. (Shenzhen, China).

### Flow cytometric analysis

To monitor the progress of selection, as well as to investigate the binding affinity and specificity of aptamer candidates, the FITC-labeled evolved ssDNA pool, selected aptamers or initial ssDNA library were incubated with 3 × 10^5^ 5-8F cells or NP69 cells in 200 μL of binding buffer containing 10% FBS on ice for 0.5 h. The final concentration of ssDNA pool, selected aptamers and initial ssDNA library was 250 nM. Cells were washed three times with 400 μL of washing buffer and resuspended in 400 μL of washing buffer. The cell samples were then analyzed by FACScan cytometer (BD Immunocytometry Systems).

To calculate the equilibrium dissociation constant (*K_d_*) of aptamers S3, 5-8F cells were incubated with different concentrations of FITC-labeled aptamers or initial ssDNA library in binding buffer containing 10% FBS on ice for 0.5 h. After incubation, cells were washed three times with washing buffer and then resuspended in 400 μL of binding buffer for flow cytometric analysis. After subtracting the mean fluorescence background of DNA library from the corresponding mean fluorescence intensity of the aptamers, the *K_d_* value of aptamer S3 was obtained by fitting the dependence of fluorescence intensity of specific binding on the concentration of aptamer to the one-site saturation equation Y = *Bmax* ·*X*/(*K_d_*+X). All of the experiments for the binding assay were repeated three times.

### Fluorescence imaging of 5-8F cells with aptamer S3

For fluorescence imaging, 1 × 10^5^ 5-8F cells were seeded on coverslip in six-well cell culture plate. After 24 h, 250 nM of the FITC-labeled aptamer or unselected FITC-labeled ssDNA library was incubated with 5-8F cells on ice for 0.5 h and washed for three times. Then the cells were fixed with 4% paraformaldehyde and washed again. At last, the cells were imaged with Nikon fluorescence microscope.

### Fluorescence imaging tumor spheres with aptamer S3

Two thousands of 5-8F cells were seeded into the wells of ultralow-attachment plates and cultured in DMEM/F12 media (Life Technologies) containing 10 ng/mL EGF, 10 ng/mL FGF and 100 units/mL B27. At the 10th day, the tumor spheres were washed and incubated with biotin-labeled aptamer or biotin-labeled initial ssDNA library on ice for 30 min. Then the spheres were incubated with streptavidin-PE for another 30 min at room temperature. To show the nucleus, the spheres were stained with Hoechst33342 for 10 min. After being washed three times, the tumor spheres were imaged with fluorescence microscope.

### Effects of temperature and proteinase treatment on aptamer binding

To investigate the effect of temperature on aptamer binding, 5-8F cells were detached using 0.02% EDTA and incubated with S3 on ice or at 37°C respectively.

5-8F cells were washed and detached by 0.25% trypsin, 0.1% proteinase K or non-enzymer buffer, respectively. After being washed for three times with washing buffer, the cells were used for binding assays as described above. The unselected FITC-labeled ssDNA library was used as a negative control.

### Staining of human NPC tissue sections using selected aptamer

All samples were obtained from patients before treatment at Hunan Cancer Hospital (Changsha, Hunan, China) with their informed consent. The formalin-fixed paraffin-embedded (FFPE) tissue sections were heated at 60°C for 1 h and dewaxed in xylene twice and each for 15 min, rehydrated though a degraded ethanol series (100%, 95%, 90%, 80% and 70% ethanol for 5, 5, 1, 1 and 1 min, respectively). After three washes with PBS for three times (3 × 3 min), the slides were heated in 0.01 M citrate buffer (pH 6.0) at 95°C for 20 min to retrieve antigens. After cooling down at room temperature, the slides were washed with PBS for three times. Before staining, the slides were blocked with binding buffer containing 0.1 mg/mL salmon sperm DNA at 4°C for 1 h and then incubated with biotin-labeled aptamer at 4°C for another 1 h. After wash three times, the slides were incubated with streptavidin-QD625 at room temperature for 0.5 h. Finally, the stained slides were imaged with Nikon fluorescence microscope after wash three times. The study was carried out after approval by the Ethics Committee of Central South University. The methods were carried out in accordance with the approved guidelines.

### Recognition of the NPC cells in real peripheral blood samples by selected aptamer

To investigate the feasibility of the selected aptamer to recognize cancer cells in real biological samples, 1 × 10^5^ of 5-8F cells or A549 cells were added into 1 mL of peripheral blood samples which were collected from healthy volunteers. Meanwhile, to test the sensitivity of aptamer, different amounts of 5-8F cells (10^5^, 10^4^, 10^3^ and 10^2^) were dispersed in 1 mL of whole blood. After treated with red cell lysis buffer (Tiangen Biotech), the mixed cells (mainly WBC and 5-8F cells) were incubated with FITC-labeled aptamer and CD45-PE (Miltenyi Biotec) on ice for 0.5 h. The samples were analyzed by flow cytometry after incubation and being washed.

### Affinity purification of target membrane protein with S3

Cells were lysed with RIPA lysis buffer containing protease inhibitor cocktail (Thermo) on ice for 30min. After centrifugation at 4°C with the speed of 13000 rpm, the supernatant was collected and proteins (2 mg) were incubated with biotin-labeled aptamer S3 and ssDNA library respectively at 4°C for 30 min. The protein-aptamer complexes were incubated with streptavidin-coated sepharose beads for another 30 min at 4°C. Then the collected beads were washed six times with cool PBS and mixed with 30 μL of 2 × SDS loading buffer, boiled, analyzed by 12% SDS-PAGE and stained with Coomassie brilliant Blue R250. The aptamer-specific protein bands were excised and trypsin-digested *in situ* and analyzed by QSTAR LC-MS/MS and MASCOT database search at the Protein Chemistry Core Facility.

### Forced expression of CD109 in 293T cells

The ORF sequence of CD109 was amplified from 5-8F cDNA and cloned into pcDNA3.1(+) vector. 293T cells were transiently transfected with plasmids by Lipofectamine 2000^TM^ reagent (Life Technologies) according to previous literature [42]. The expression level of CD109 after transiently transfection was detected by Western blot. The binding between aptamer S3 and the CD109-expressed 293T cells was analyzed by flow cytometry.

### Western blot

Western blot was done in accordance with our manual [43]. In brief, cells were lysed by RIPA and total proteins were obtained. After high-speed centrifugation, the proteins were denatured. Forty micrograms of protein was loaded and separated by SDS-PAGE. The samples were transferred to PVDF membrane (Millipore) followed by immunoblotting with anti-CD109 sheep polyclonal antibody (R&D) and anti-β-actin mouse monoclonal antibody (Sigma) respectively after block. At last, Gel Imaging System (Bio-Rad) was used to visualize the protein bands after incubation with horseradish peroxidase (HRP)-conjugated anti-IgG antibody.

### Immunohistochemistry (IHC) experiment

Tissue slides were immunoreacted with anti-CD109 sheep polyclonal antibody and detected with SAB system (DAKO, Carpinteria, CA). Sections were independently evaluated and scored by two pathologists who were blind to clinical data. Evaluation of staining was assessed using the immunoreactive score (IRS), according to previous literature [44].

### Electrophoretic mobility shift assay (EMSA)

The EMSA assay was used to show the specificity of S3-CD109 binding in vitro. We incubated recombinant human CD109 protein (R&D) and FAM-labeled S3 at 4°C while shaking in binding buffer. After incubation, samples were taken out and loaded with loading buffer in 5% native PAGE in 0.5×TBE. The unselected FITC-labeled ssDNA library was used as a negative control.

### Quantitative reverse transcription-PCR (qPCR)

QPCR reactions were performed as described [45]. And glyceraldehyde-3-phosphate dehydrogenase (GAPDH) was used as an internal control. The primers were listed as follow: CD109 forward primer 5′- GAAGCCATCTCTCAACTTCACA-3′, CD109 reverse primer 5′- TTCCACTGTTAGATCCGCTCC-3′, GAPDH forward primer 5′-ACCACAGTCCATGCCAT CAC-3′, GAPDH reverse primer 5′- TCCACCACCCT GTTGCTGT -3′.

## SUPPLEMENTARY MATERIALS


