# Supplementary material for: CD109 is identified as a potential nasopharyngeal carcinoma biomarker using aptamer selected by cell-SELEX
Source: Oncotarget. 2016 Jul 11;7(34):55328–42. doi: 10.18632/oncotarget.10530 (PMC5342420; doi:10.18632/oncotarget.10530)
Supplement: Supplementary file 1 [file oncotarget-07-55328-s001.pdf]

CD109 is identified as a potential nasopharyngeal carcinoma biomarker using aptamer selected by cell-SELEX

SUPPLEMENTARY MATERIAL

S3

5’-ATCCAGAGTGACGCAGCATCTGAGAATAGTGGTTTGCTGTATGGTGGGCGTTGAAAGA GGGGTGGACACGGTGGCTTAGT-3’

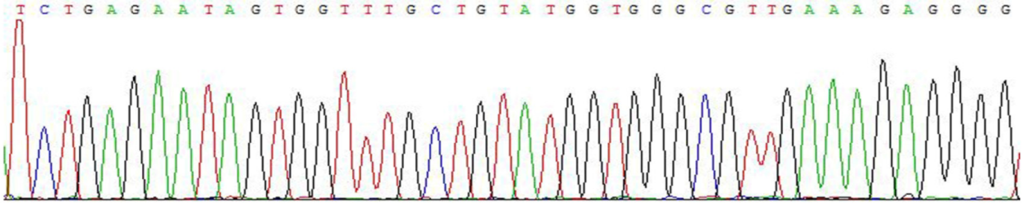

S5

5’-ATCCAGAGTGACGCAGCACTGTGGCGGGATTCTGGCAAAGTTTCGAGCCCTGGTAAG AGTGTGGACACGGTGGCTTAGT-3’

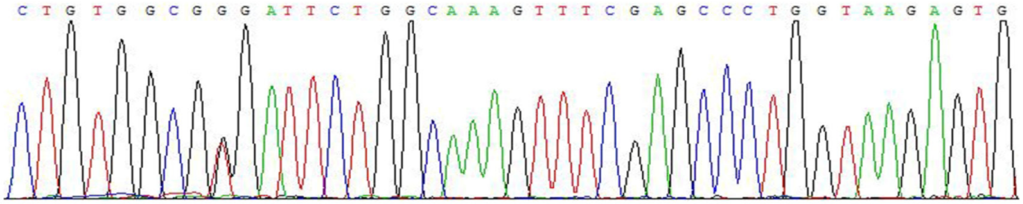

S12

5’-ATCCAGAGTGACGCAGCACGCCGTAGTATGGTGCAGATGGTTTGCTGTATGGTGGGCG CACCTGGACACGGTGGCTTAGT-3’

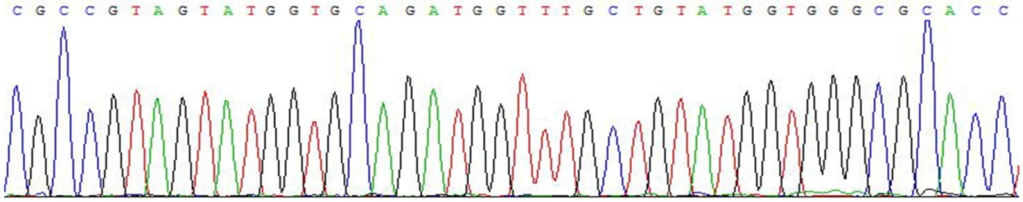

S27

5’-ATCCAGAGTGACGCAGCAGAAAGTAAGGGTAGTTTGGGGGCTCGTATGGGGGGAGGTT CTGATGGACACGGTGGCTTAGT-3’

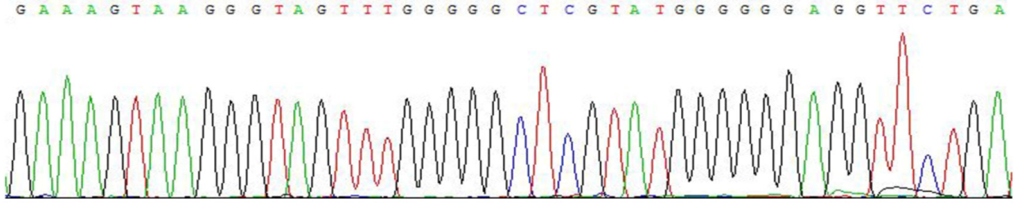

Supplementary Figure S1: Sequencing of selected aptamers (S3, S5, S12, S27).

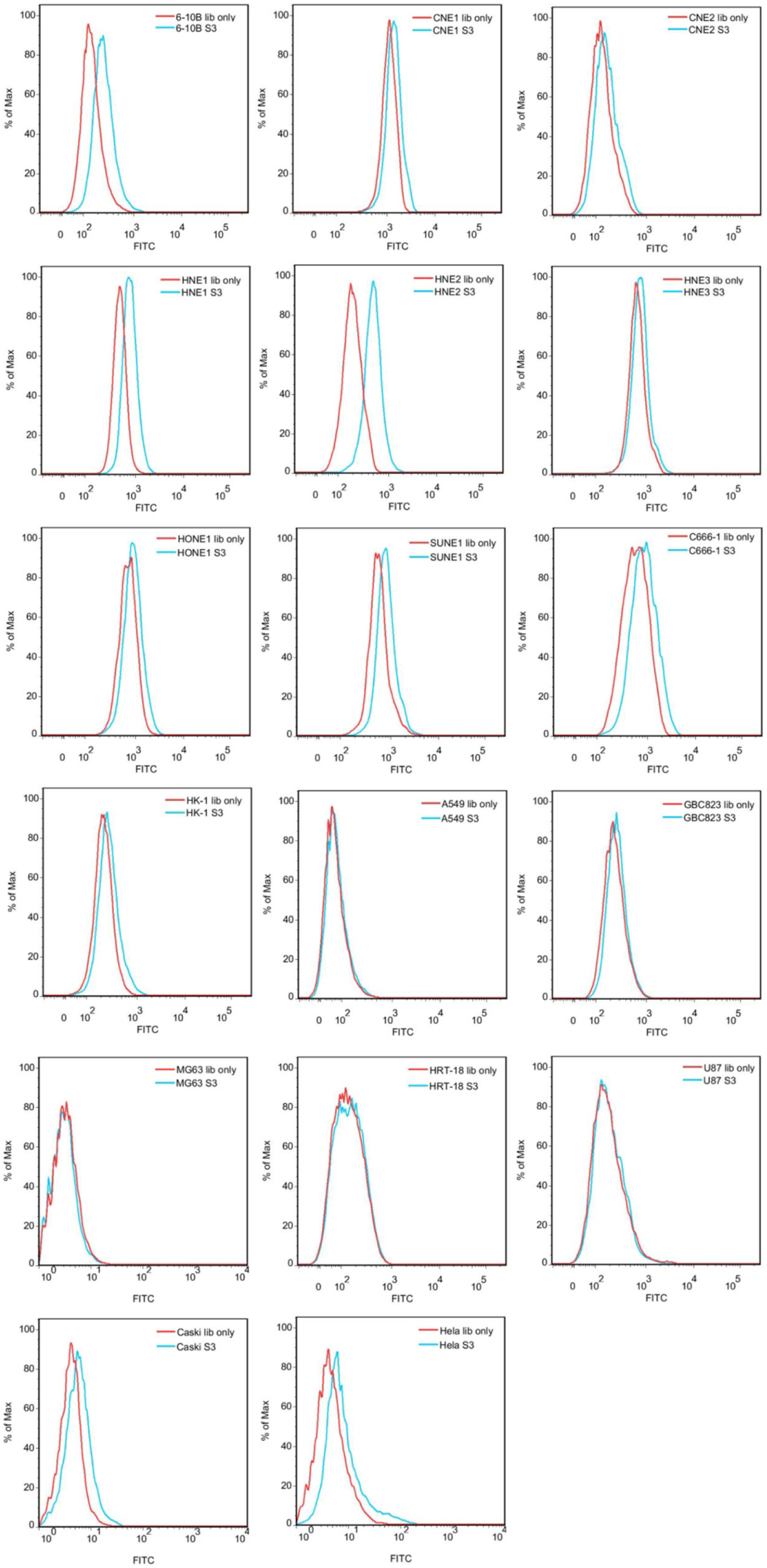

Supplementary Figure S2: Binding ability of aptamer S3 on different cell lines. The unselected initial library was used as control.
